# HOOK3 suppresses proliferation and metastasis in gastric cancer via the SP1/VEGFA axis

**DOI:** 10.1038/s41420-024-01808-8

**Published:** 2024-01-16

**Authors:** Kexi Yang, Juntao Li, Jinghan Zhu, Yuqi Chen, Yuxin He, Jiayu Wang, Kanger Shen, Kun Wang, Tongguo Shi, Weichang Chen

**Affiliations:** 1 Jiangsu Institute of Clinical Immunology, The First Affiliated Hospital of Soochow University, Suzhou, China; 2 Jiangsu Key Laboratory of Clinical Immunology, Soochow University, Suzhou, China; 3 Department of Gastroenterology, The First Affiliated Hospital of Soochow University, Suzhou, China; 4 Department of Gastroenterology, Dushu Lake Hospital Affiliated of Soochow University, Suzhou, China

**Keywords:** Gastrointestinal cancer, Cell growth, Cell migration

## Abstract

HOOK3, a member of the human hook microtubule-tethering protein family, has been implicated in the progression of cancer. However, the role of HOOK3 in the pathogenesis of gastric cancer (GC) remains incompletely understood. In this study, we investigated the expression of HOOK3 protein in GC tissues using immunohistochemistry (IHC). The findings of our study indicate that the expression levels of HOOK3 in GC tissues were relatively low. Furthermore, a significant negative association was seen between HOOK3 expression and the prognosis of patients with GC. The suppression of HOOK3 resulted in a notable increase in the proliferation, migration, invasion, and survival of GC cells. Conversely, the overexpression of HOOK3 had the opposite impact, reducing these cellular processes. Moreover, in vivo tests have shown evidence that the overexpression of HOOK3 significantly inhibited the formation of tumors and the spread of GC cells to the lungs. In a mechanistic manner, the analysis of RNA-seq data demonstrated that the knockdown of HOOK3 resulted in a notable increase in the expression of vascular endothelial growth factor A (VEGFA) in GC cells. Furthermore, the upregulation of VEGFA counteracted the impacts of HOOK3 upregulation on the proliferation, migration, invasion, and survival of GC cells. Furthermore, it was revealed that specificity protein 1 (SP1) exhibited the ability to bind to the promoter region of VEGFA. Moreover, the overexpression of SP1 successfully counteracted the inhibitory impact of HOOK3 overexpression on the expression of VEGFA in GC cells. In summary, the results of our study indicate that HOOK3 has a role in inhibiting the growth, migration, invasion, and survival of GC cells by modulating the SP1/VEGFA pathway. These findings contribute significant knowledge to our understanding of the underlying mechanisms involved in the development of GC.

## Introduction

On the basis of the latest GLOBOCAN report, there were 1,089,000 new cases of gastric cancer (GC) in 2020, with 769,000 deaths related to GC [1]. Consequently, it ranks as the forth most common cause of cancer-related deaths [2]. The prognosis of patients with GC has improved through various methods such as endoscopy, surgery, and standard chemotherapy. However, patients with advanced stages of GC and metastasis still face an unfavorable prognosis. Hence, there is a pressing need to explore new targets for the diagnosis and treatment of GC.

Hook microtubule-tethering protein 3 (HOOK3) is a member of human hook microtubule-tethering protein family, initially reported in 2001 [3]. HOOK3 is reported to be an adaptor protein that connects organelles with microtubules and is involved in the structure and localization of the Golgi complex [3]. In recent years, there has been evidence suggesting a correlation between HOOK3 and the development of various types of malignancies, including prostate cancer, myelodysplastic syndrome (MDS), non-small cell lung cancer, and papillary thyroid carcinoma [4–7]. For example, high-level HOOK3 protein expression was positively associated with advanced tumor stage and high proliferation index in prostate cancer [7]. And HOOK3 expression was demonstrated to be an independent predictor of poor prognosis in prostate cancer [7]. Xuehong Zhang et al. showed that the HOOK3-FGFR1 fusion gene was involved in the pathogenesis of MDS and activated the NF-kappaB pathway [5]. However, the expression and biological functions of HOOK3 in GC remain unclear.

In this study, we attempted to investigate the expression of HOOK3 in patients with GC and the roles in modulating GC progression. It was revealed that the protein expression of HOOK3 exhibited a considerable downregulation in the tumor tissues of patients diagnosed with GC. Furthermore, it has been observed that a decreased level of HOOK3 expression is indicative of an unfavorable outcome in patients diagnosed with GC. The downregulation of HOOK3 was found to enhance the proliferation, migration, invasion, and survival of GC cells via activating the SP1/ VEGFA pathway. Conversely, the overexpression of HOOK3 was observed to suppress these cellular processes. Furthermore, the overexpression of HOOK3 exhibited inhibitory effects on tumor growth and lung metastasis in an in vivo setting. In conclusion, we have successfully identified and demonstrated the significant involvement of HOOK3 in the regulation of GC progression. This finding offers valuable insights into the pathophysiology of GC.

## Results

### HOOK3 is lowly expressed in GC tissues and its low expression predicts a poor prognosis

**Fig. 1:**
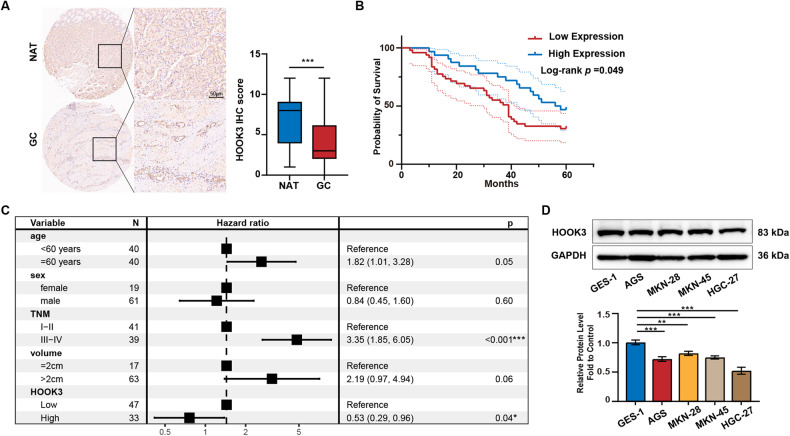
HOOK3 was decreased in GC tissues and negatively related to poor prognosis in GC patients. **A** Total of 81 GC samples and 70 normal adjacent tissues were subjected to immunohistochemistry (IHC) analysis to assess the expression of HOOK3. An illustrative image representing the results is provided. **B** The overall survival of GC patients was stratified based on high and low HOOK3 expression groups, and Kaplan-Meier curves were generated. **C** Multivariate Cox regression analysis was conducted to evaluate the association between HOOK3 expression and overall survival, as depicted in the forest plots. **D** Western blotting was employed to measure HOOK3 protein expression in GES-1, AGS, HGC27, MKN28, and MKN45 cells, with GAPDH serving as a loading control. The Image J program was utilized for the quantification of Western blot band densities. The resulting values were presented as means with standard deviations (SD), and statistical significance was evaluated using Student’s *t*-test. **P < 0.01, and ***P < 0.001.

According to the data shown in Fig. 1A, the protein expression of HOOK3 in GC tissue demonstrated a notable reduction in comparison to normal gastric tissue. Moreover, the analysis of HOOK3 expression revealed a statistically significant association with the level of differentiation in GC, as indicated in Table 1. As a result, a study was performed to examine the correlation between the expression of HOOK3 and the prognosis of individuals diagnosed with GC. The data shown in Fig. 1B indicates that individuals with low HOOK3 expression exhibited a significantly reduced survival rate compared to those with high HOOK3 expression in GC. The results of the multivariate Cox regression analysis demonstrated that both the TNM stage and low HOOK3 expression were identified as significant risk factors for the overall survival of patients with GC (Fig. 1C). Furthermore, it was noted that the expression levels of HOOK3 were comparatively lower in GC cell lines (AGS, MKN-28, MKN-45, and HGC-27) in comparison to the gastric mucosal epithelial cell line GES-1, as depicted in Fig. 1D.

**Table 1:** HOOK3 expression and clinical features in 81 gastric cancer patient samples.

Variables	Low HOOK3 (*n* = 48)	High HOOK3 (*n* = 33)	*P* value
Sex			
Female	10	9	0.502
Male	38	24	
Age			
<60	28	13	0.094
≥60	20	20	
TNM stage			
I-II	23	19	0.393
III-IV	25	14	
Tumor differentiatinon			
Well and moderately differentiated	14	22	0.001
Poorly differentiated	34	11	
Tumor size (cm)			
≤2	9	8	0.584
>2	38	25	

### HOOK3 knockdown promoted proliferation, migration, invasion, and survival in GC cells

**Fig. 2:**
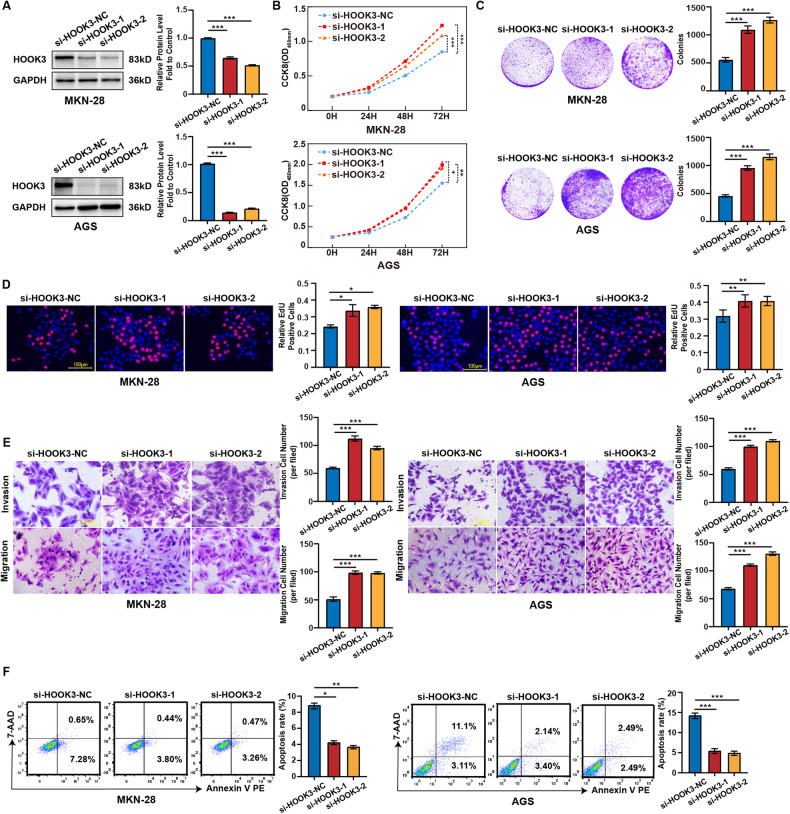
HOOK3 knockdown promoted the proliferation, migration, and invasion in GC cells. **A** The protein expression of HOOK3 in MKN-28 and AGS cells was examined following transfection with si-HOOK3-1 or si-HOOK3-2. GAPDH was used as a loading control. The densities of Western blot bands were quantified using the ImageJ program. **B** The proliferation of AGS and MKN-28 cells was assessed after transfection with si-HOOK3-1 or si-HOOK3-2 using a CCK-8 assay. **C** Colony formation assays were performed on AGS and MKN-28 cells transfected with si-HOOK3-1 or si-HOOK3-2. **D** EdU analysis was conducted to evaluate the proliferative ability of AGS and MKN-28 cells treated with si-HOOK3-1 or si-HOOK3-2. Representative images were provided, and a scale bar of 100 μm was included. The bar graph shows the statistical analysis of the percentage of EdU-positive cells in transfected GC cells. **E** Transwell migration and invasion assays were conducted on AGS and MKN-28 cells transfected with si-HOOK3-1 or si-HOOK3-2. **F** Apoptosis assays were conducted were performed on AGS and MKN-28 cells transfected with si-HOOK3-1 or si-HOOK3-2. The experiments were performed in triplicate. The data are presented as the mean with standard deviation (SD), and statistical significance was determined using Student’s *t*-test. *P < 0.05, **P < 0.01, and ***P < 0.001.

In order to gain a deeper understanding of the biological role of HOOK3, we performed experiments aimed at suppressing the expression of HOOK3 in AGS and MKN-28 cells. Consequently, a notable reduction in the levels of HOOK3 protein was detected after to knockdown, as depicted in Fig. 2A. In addition, we conducted cell counting (CCK8) and colony formation experiments to evaluate the proliferation rate of AGS and MKN-28 cells following HOOK3 knockdown. The results indicated a significant enhancement in cell proliferation, as depicted in Fig. 2B, C. The results obtained from the EdU studies provided additional support for these conclusions (Fig. 2D). Furthermore, the findings from transwell tests provided confirmation that the downregulation of HOOK3 resulted in enhanced migratory and invasion capabilities of AGS and MKN-28 cells (Fig. 2E). Moreover, the downregulation of HOOK3 led to a significant decrease in the apoptosis rate of MKN-28 and AGS cells (Fig. 2F).

### HOOK3 overexpression inhibited proliferation, migration, invasion, and survival in GC cells

**Fig. 3:**
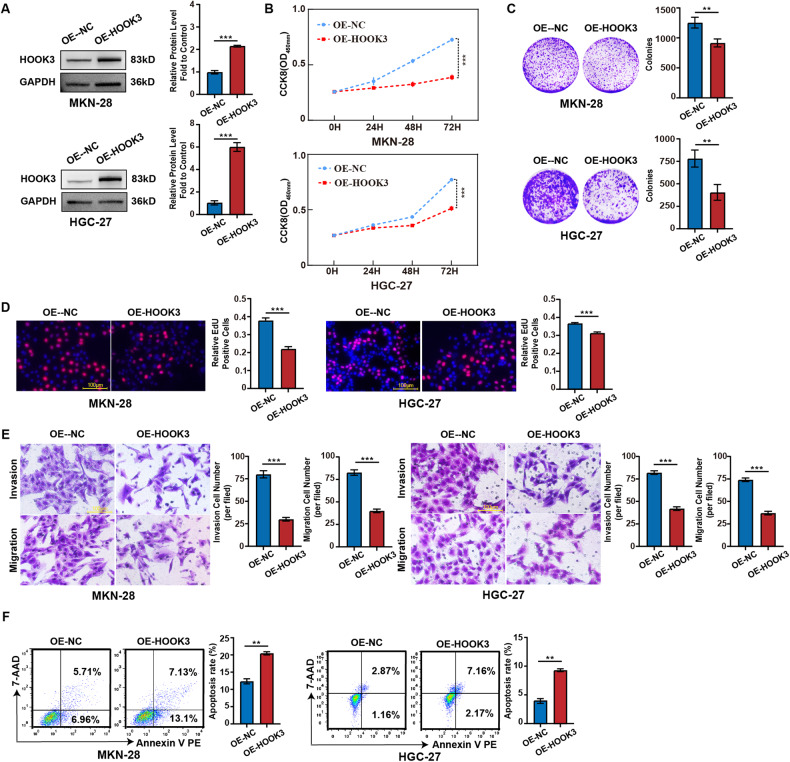
HOOK3 overexpression inhibited the proliferation, migration, and invasion in GC cells. **A** The protein expression of HOOK3 was examined in MKN-28 and HGC-27 cells with stable overexpression of HOOK3. GAPDH was used as a loading control. The quantification of band densities in Western blot analysis was performed using the ImageJ program. **B** The proliferation of MKN-28 and HGC-27 cells with stable overexpression of HOOK3 was assessed using a CCK-8 assay. **C** The colony formation assay was conducted to evaluate the ability of HOOK3 overexpressing MKN-28 and HGC-27 cells to form colonies. **D** EdU analysis was carried out to measure the proliferative ability of MKN-28 and HGC-27 cells with HOOK3 overexpression. Representative images are presented, with a scale bar of 100 μm. The percentage of EdU-positive cells in transfected GC cells was statistically analyzed and shown in the bar graph. **E** Transwell migration and invasion assays were performed to examine the migratory and invasive capacities of MKN-28 and HGC-27 cells with HOOK3 overexpression. **F** Apoptosis assays were performed on MKN-28 and HGC-27 cells with HOOK3 overexpression. The experiments were conducted in triplicate. The data were represented as means with standard deviation (SD), and statistical significance was assessed using Student’s *t*-test. **P < 0.01, and ***P < 0.001.

Subsequently, stable cell lines of MKN-28 and HGC-27 were generated by infecting them with lentivirus carrying the HOOK3 overexpression construct. Based on the data depicted in Fig. 3A, a notable elevation in the expression levels of the HOOK3 protein was seen in MKN-28 and HGC-27 cells that underwent stable overexpression of HOOK3. The experimental findings derived from CCK8, colony formation, and EdU assays provided evidence that the overexpression of HOOK3 resulted in a significant decrease in the proliferation rate of MKN-28 and HGC-27 cells, as illustrated in Fig. 3B–D. Furthermore, the overexpression of HOOK3 was found to inhibit the migration and invasion of MKN-28 and HGC-27 cells, as demonstrated in Fig. 3E. Moreover, the overexpression of HOOK3 significantly upregulated the apoptosis rate of MKN-28 and HGC-27 cells (Fig. 3F).

### VEGFA is involved in HOOK3-mediated proliferation, migration, invasion, and survival

**Fig. 4:**
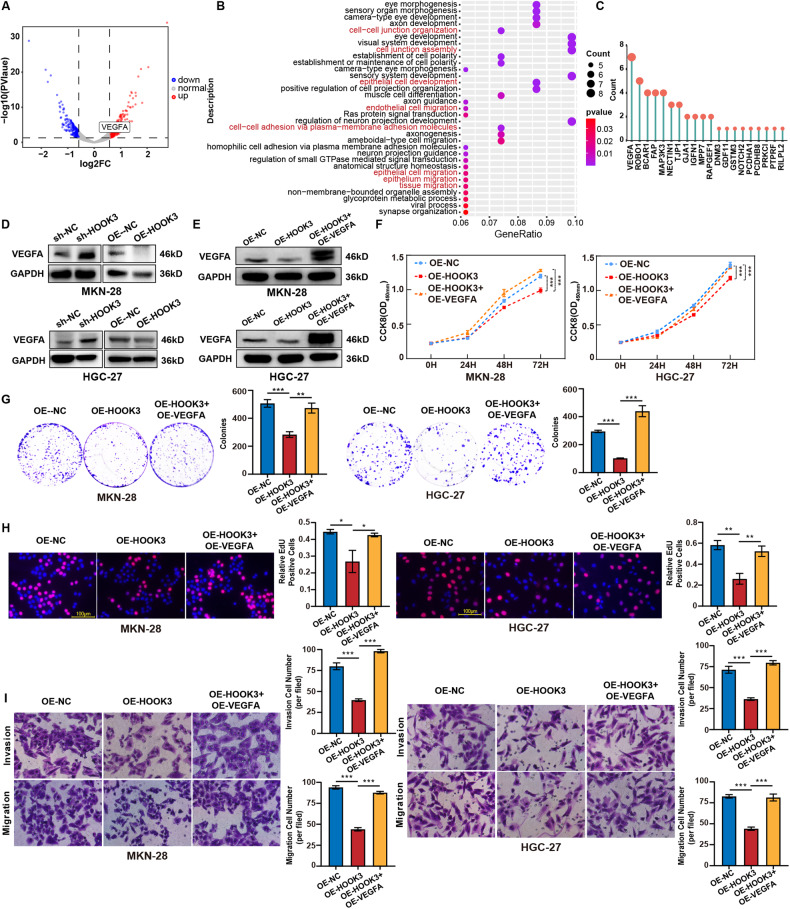
VEGFA was involved in HOOK3-mediated proliferation, migration, and invasion in GC cells. **A** Volcano plot of differentially expressed genes (DEGs) in MKN-28 cells and HOOK3-knockdown MKN-28 cells. **B** Gene Ontology (GO) enrichment analysis of the top 0.5% negatively correlated genes among the DEGs. **C** The frequency of genes related to migration and invasion observed in the GO enrichment analysis. **D** The protein levels of VEGFA were measured in HOOK3-overexpressing MKN-28 and HGC-27 cells, as well as HOOK3-knockdown MKN-28 and HGC-27 cells, with GAPDH serving as a loading control. **E** The protein levels of VEGFA were assessed in HOOK3-overexpressing MKN-28 and HGC-27 cells treated with a plasmid that overexpresses VEGFA, with GAPDH used as a loading control. **F** The proliferation of MKN-28 and HGC-27 cells overexpressing HOOK3 and treated with a plasmid overexpressing VEGFA was evaluated using a CCK-8 assay. **G** Colony formation assays were performed on MKN-28 and HGC-27 cells with HOOK3 overexpression and treated with a VEGFA-overexpressing plasmid. **H** EdU analysis was conducted on MKN-28 and HGC-27 cells overexpressing HOOK3 after treatment with a plasmid that overexpresses VEGFA. **I** Transwell migration and invasion assays were carried out on HOOK3-overexpressing MKN-28 and HGC-27 cells treated with a VEGFA-overexpressing plasmid. The experiments were conducted in triplicate. The values were represented as means with standard deviation (SD), and statistical significance was determined using Student’s *t*-test. *P < 0.05, **P < 0.01, and ***P < 0.001.

To explore the probable processes behind the regulation of the malignant phenotype of GC by HOOK3, we performed RNA-seq study on MKN-28 cells in which HOOK3 was knocked down, as well as a control group. Based on the findings obtained from RNA-seq analysis, a total of 515 genes were identified as differently expressed in cells overexpressing HOOK3 (Table S2). Among these genes, 305 were found to be upregulated, while 210 were downregulated. These differential expression patterns were determined based on a fold change threshold of greater than 0.5 and a statistical significance level of P-value less than 0.05, as depicted in Fig. 4A. Following this, a correlation analysis was conducted to examine the expression levels of HOOK3 in connection to other genes. From this analysis, the top 0.5% of genes that exhibited a negative correlation with HOOK3 were selected for further enrichment analysis (Table S3). The analysis demonstrated that a majority of these genes exhibited enrichment in functions associated with migration and invasion. Notably, VEGFA emerged as the gene most commonly detected in this context (Fig. 4B, C). Moreover, an inverse correlation was seen between the mRNA expression levels of HOOK3 and VEGFA, as indicated by the analysis of data obtained from the TCGA database (Supplementary Fig. 1A). The confirmation of the negative association between HOOK3 and VEGFA was achieved through the implementation of Western blot investigations, which involved the analysis of protein expression levels for both HOOK3 and VEGFA (Fig. 4D, Supplementary Fig. 2A, B). To explore the potential biological significance of HOOK3 in relation to VEGFA, we inserted a plasmid containing VEGFA overexpression into MKN-28 and HGC-27 cells that already exhibit stable overexpression of HOOK3. Based on the data depicted in Fig. 4E, it can be observed that the protein expression of VEGFA exhibited an elevated level in stable HOOK3 overexpressing GC cells that underwent transfection with a plasmid overexpressing VEGFA, in comparison to stable HOOK3 overexpressing GC cells. In addition, it was observed that the upregulation of VEGFA counteracted the effects of HOOK3 upregulation on the proliferation of MKN-28 and HGC-27 cells, as depicted in Fig. 4F–H. Furthermore, it was observed in Fig. 4I that the inhibitory effects of HOOK3 on the migration and invasion of GC cells were counteracted by the overexpression of VEGFA. Moreover, the upregulation of VEGFA counteracted the effects of HOOK3 upregulation on the apoptosis of MKN-28 and HGC-27 cells (Supplementary Fig. 3A). Besides, it was noted that the conditioned medium obtained from OE-HOOK3 GC cells led to a significant reduction in the number of branch points, a marker of human umbilical vein endothelial cells (HUVEC) angiogenesis. In contrast, the OE-HOOK3 GC cells transfected with VEGFA overexpressing plasmids demonstrated a reversal of this phenomenon (Supplementary Fig. 3B). The findings of this study suggest that the involvement of HOOK3 in the regulation of VEGFA contributes to the suppression of malignant traits in GC.

### HOOK3 inhibits VEGFA expression via SP1

**Fig. 5:**
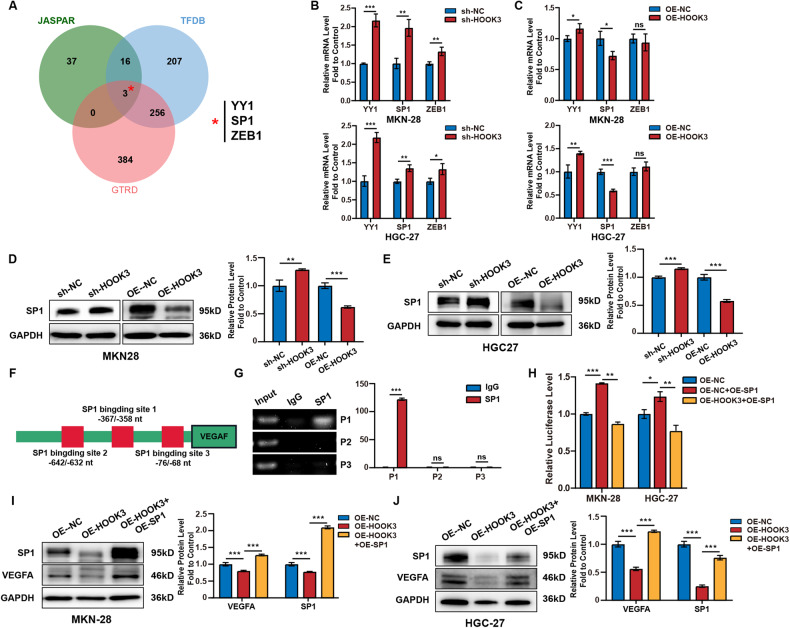
HOOK3 inhibited VEGFA expression via SP1. **A** The intersection of transcription factors predicted by three databases, GTRD (red circle), JASPAR (green circle), and TFDB (blue circle), was analyzed. **B** The expression of YY1, SP1, and ZEB1 was analyzed by RT-qPCR in HOOK3 knockdown MKN-28 and HGC-27 cells. **C** The expression of YY1, SP1, and ZEB1 was analyzed by RT-qPCR in HOOK3-overexpressing MKN-28 and HGC-27 cells. **D** Western blot analysis was performed to evaluate the expression of SP1 in either HOOK3 knockdown or HOOK3-overexpressing MKN-28 cells. GAPDH was used as the control. The band densities from Western blot were quantified using the ImageJ program. **E** Western blot analysis was performed to evaluate the expression of SP1 in either HOOK3-knockdown or HOOK3-overexpressing HGC-27 cells. GAPDH was used as the control. The band densities from Western blot were quantified using the ImageJ program. **F** A schematic diagram illustrating the presence of three SP1 binding sites (P1: −367nt to −358nt, P2: −642nt to −632nt, and P3: −76nt to −68nt) in the 5′ region of the VEGFA promoter. **G** ChIP analysis was conducted to assess the binding of SP1 to the VEGFA promoter in MKN-28 cells. Normal rabbit IgG was used as the control. **H** The luciferase activity, responsive to SP1, was measured in MKN-28 and HGC-27 cells transfected with HOOK3 overexpressing plasmid and SP1 overexpressing plasmid. **I** Western blot analysis was performed to evaluate the expression of VEGFA and SP1 in HOOK3-overexpressing MKN-28 cells transfected with SP1 overexpression plasmid. GAPDH was used as the control. The band densities from Western blot were quantified using the ImageJ program. **J** Western blot analysis was performed to evaluate the expression of VEGFA and SP1 in HOOK3-overexpressing HGC-27 cells transfected with SP1 overexpression plasmid. GAPDH was used as the control. The band densities from Western blot were quantified using the ImageJ program. The experiments were conducted in triplicate. The mean values with standard deviation (SD) were presented, and the statistical significance was determined using Student’s *t*-test. Nonsignificant results were denoted as “ns”, while significance levels were shown as *P < 0.05, **P < 0.01, and ***P < 0.001.

Our study aimed to examine the regulatory role of HOOK3 in the modulation of VEGFA expression in GC cells. Based on the anticipated outcomes derived from the JASPAR, TFDB, and GTRD databases, our analysis reveals the presence of three putative transcription factors, namely YY1, SP1, and ZEB1, inside the promoter region of VEGFA (Fig. 5A). The findings from the RT-qPCR analysis, as depicted in Fig. 5B, C, indicate a negative correlation between HOOK3 and SP1 in both MKN-28 and HGC-27 cell lines. Furthermore, the knockdown of HOOK3 resulted in an increase in the protein expression of SP1 in MKN-28 and HGC-27 cells, as observed in Fig. 5D, E. In contrast, the upregulation of HOOK3 resulted in a reduction in the protein expression of SP1 within the identical cell lines. The results of this study indicate that HOOK3 potentially plays a function in the modulation of VEGFA synthesis in GC cells by interacting with SP1. In order to validate this idea, ChIP assays were conducted to investigate the potential binding of SP1 to the promoter region of VEGFA. The VEGFA promoter had three binding sites for SP1, namely P1, P2, and P3. The ChIP assay findings demonstrated a high enrichment of DNA in P1 when purified with the anti-SP1 antibody (Fig. 5F, G). Furthermore, the luciferase reporter gene test demonstrated a clear enhancement in the activity of SP1 due to its overexpression, as evidenced by the increased SP1 DNA-binding activity. However, this effect was reversed when HOOK3 was overexpressed, as depicted in Fig. 5H. The Western blot examination yielded findings indicating that the introduction of the SP1 expression plasmid resulted in a notable augmentation in the protein abundance of SP1. The observed augmentation effectively counteracted the suppressive effect of HOOK3 overexpression on the production of VEGFA in both MKN-28 and HGC-27 cells, as illustrated in Fig. 5I, J. The results of this study suggest that HOOK3 has a suppressive impact on the synthesis of VEGFA in GC cells through inhibiting the expression of SP1.

### HOOK3 overexpression repressed growth and metastasis of GC in vivo

**Fig. 6:**
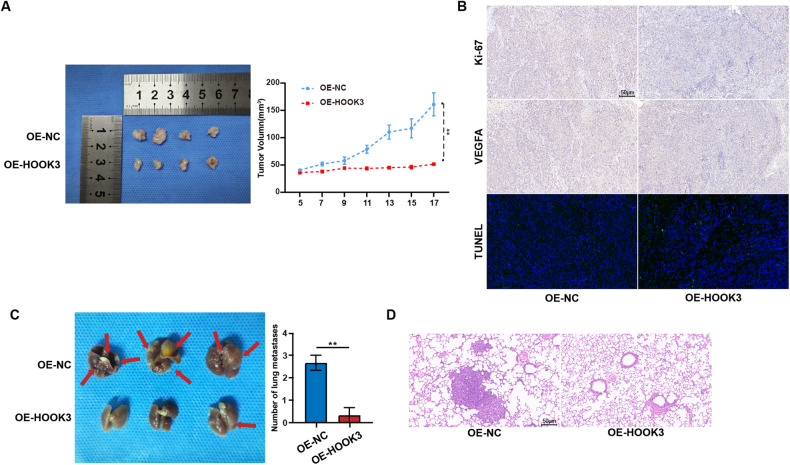
HOOK3 overexpression suppressed proliferation and metastasis of GC cells in vivo. **A** The image displayed is representative of HOOK3-overexpressing MKN28 tumors grown in nude mice. The volumes of these tumors were evaluated in groups containing four mice each. **B** Representative images of immunohistochemistry (IHC) staining for Ki-67 and VEGFA and TUNEL staining were captured in tumor tissues from the OE-HOOK3 and OE-NC groups. The scale bar represents 50 μm. **C** A murine lung metastasis model was established using HOOK3-overexpressing GC cells and control GC cells, with three mice in each group. **D** Representative images of hematoxylin and eosin (HE) stains were obtained from lung metastasis in the OE-HOOK3 and OE-NC groups. The scale bar represents 50 μm. The mean values along with standard deviations (SD) were used to represent the data, and statistical significance was determined using Student’s *t*-test. **P < 0.01.

In order to evaluate the influence of HOOK3 on the proliferation of GC tumors in vivo, we established xenograft mouse models by introducing stable overexpression of HOOK3 in MKN-28 cells. Through the utilization of tumor imaging techniques and the measurement of tumor volume, our findings unequivocally illustrated that the overexpression of HOOK3 had a substantial inhibitory effect on tumor growth in vivo (Fig. 6A). Furthermore, in comparison to the control group, the xenograft tissues from the HOOK3 overexpression group demonstrated a significant reduction in the expression levels of Ki67 and VEGFA (Fig. 6B). Besides, xenograft tissues from the HOOK3 overexpression group exhibited a markedly increase in positive Terminal deoxynucleotidyl transferase dUTP nick-end labeling (TUNEL) staining compared with tumors derived from control group (Fig. 6B). In order to conduct a more comprehensive investigation into the potential role of HOOK3 in the in vivo metastasis of GC, we created a mouse model of lung metastasis by introducing MKN-28 cells that had been genetically modified to overexpress HOOK3. The results presented in Fig. 6C, D demonstrate that the overexpression of HOOK3 significantly reduced the metastatic potential of these cells in the lungs of mice. There was a notable decrease in the quantity of metastatic lesions observed in the OE-HOOK3 group.

## Discussion

This study focused on investigating the roles and molecular mechanisms of HOOK3 in the progression of GC. While a previous study has reported a positive correlation between high expression of HOOK3 and poor prognosis in prostate cancer [4], our findings revealed a significant decrease in HOOK3 expression level in GC tissues compared to adjacent non-cancerous tissues. Notably, the low expression of HOOK3 was found to be predictive of poor prognosis and overall survival in GC patients, indicating its potential as a tumor suppressor in GC. It should be noted that this study included a relatively small sample size of 81 GC patients, thus limiting the significance of our findings. Further investigations involving a larger population are warranted to elucidate the expression pattern and clinical significance of HOOK3 in GC.

The roles of HOOK3 in various cancer types exhibit inconsistency. To illustrate, a study identified a HOOK3:RET fusion in an instance of papillary thyroid cancer, demonstrating its oncogenic potential through a mouse xenograft cancer model [7]. By contrast, overexpression of HOOK3 could reverse the promoting effects of midazolam on cisplatin-sensitivity in cisplatin-resistant non-small cell lung cancer cells [6]. Herein, our results showed that overexpression of HOOK3 could inhibit the proliferation, migration, and survival of GC cells in vivo and in vitro. HOOK3 knockdown had the opposite effect. These results suggest that HOOK3 acts as a tumor suppressor in GC.

VEGF is a type of endothelial cell-specific cytokine, which includes multiple family members such as VEGFA, VEGFB [8], VEGFC [9], VEGFD [10], and PLGF [11]. Currently, VEGFA is the most extensively studied member and plays an important role in angiogenesis [12–14]. However, the function of VEGFA is not limited to influencing angiogenesis and vascular permeability; its autocrine and paracrine actions also play key roles in tumor initiation and progression. For example, VEGF acted as a survival factor and protected breast tumor cells from apoptosis by regulating the PI3K pathway, particularly under hypoxic stress [15]. In ovarian cancer, blockade of the VEGF signal resulted in increased tumor cell apoptosis, decreased proliferative index, and decreased microvessel density [16]. In order to investigate the underlying mechanism by which HOOK3 regulates the proliferation and metastasis of GC cells, we conducted RNA-seq test to identify the DEGs in MKN-28 cells that overexpress HOOK3. VEGFA garnered our attention within the pool of DEGs due to its pivotal involvement in the progression of tumors. Additional experimental data provided further confirmation of the inhibitory effect of HOOK3 on VEGFA expression, as observed at both the mRNA and Western blot levels. Furthermore, the introduction of a plasmid that overexpresses VEGFA through transfection has the potential to mitigate the suppressive impact of HOOK3 overexpression on the proliferation, migration, invasion, and survival abilities of GC cells. In conclusion, the findings of this study suggest that the HOOK3 gene has a role in suppressing the progression of GC by downregulating the expression of VEGFA.

SP1 is a transcription factor, that interacts with GC-rich promoter sequences, and it has been demonstrated to be a sequence-specific DNA-binding protein [17–19]. The majority of research have reported a greater expression level of SP1 in tumor tissues compared to normal or neighboring tissues [20–23]. Early studies have noted the regulatory relationship between SP1 and VEGFA. For example, Wang et al. found a positive correlation between the expression levels of SP1 and VEGFA in GC [24]. A study by Mathias et al. discovered the binding of the SP1/SP3 complex to the −88/−50 region of the VEGFA promoter [25]. Meng and colleagues found a binding site of SP1 in the −97/−88 region of the VEGFA promoter [26]. In this study, prediction of VEGFA binding sites was performed using online websites, and ChIP analysis showed significant enrichment of DNA precipitated by anti-SP1 antibody in the VEGFA promoter sequence. Importantly, SP1 overexpression abolished the effect of HOOK3 on VEGFA expression in GC cells, evidenced by luciferase activity assay and western blot. These data suggest that HOOK3 modulates VEGFA expression in GC cells through the inhibition of SP1. However, the mechanism by which HOOK3 regulates SP1 is currently unclear and requires further research for clarification.

**Fig. 7:**
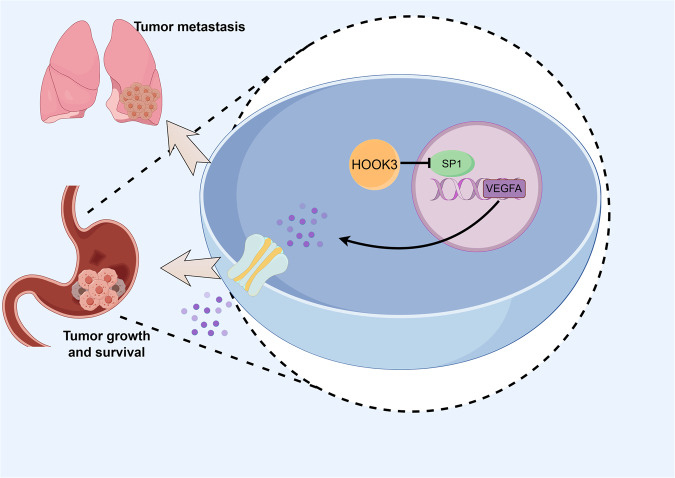
The mechanism of HOOK3 regulating VEGFA/SP1 in GC. Schematic diagram of regulation mechanism of HOOK3/VEGFA/SP1 axis in GC.

In summary, this study investigated the role and molecular mechanism of HOOK3 in the proliferation, migration, invasion and survival processes of GC. The findings of this study indicate that the upregulation of HOOK3 has a suppressive effect on the proliferation, metastasis and survival of GC through the regulation of the SP1/VEGFA pathway (Fig. 7). Hence, the strategic intervention on the HOOK3-SP1-VEGFA axis holds potential as a viable therapeutic strategy for addressing GC.

## Materials and methods

### Clinical Samples and IHC assay

The tissue microarray (TMA) utilized in this investigation was procured from Shanghai Biochip Co., Ltd, located in Shanghai, China. The study encompassed a total of 81 instances of GC tissues and 70 instances of normal adjacent tissues (NAT) tissues. The clinical data of each patient were provided in Table 1. The immunohistochemistry (IHC) assay and scoring criteria were conducted according to previously described methods. In this study, the GC TMA or paraffin-embedded mouse tumor tissues underwent deparaffinization and rehydration. Subsequently, they were subjected to incubation with specific antibodies, namely rabbit anti-HOOK3 polyclonal antibody (Proteintech, Wuhan, China, #15457-1-AP), VEGFA (Proteintech, #19003-1-AP), or Ki-67 (Proteintech, #27309-1-AP), at a temperature of 4 °C overnight. Subsequently, the tissues were subjected to incubation with a secondary antibody labeled with horseradish peroxidase (HRP) at a temperature of 37 °C for a duration of 1 h. The immunohistochemistry scores were assessed by two proficient pathologists who were unaware of the experimental conditions.

### Cell culture

The GES-1, AGS, MKN-45, MKN-28, and HGC-27 cell lines were obtained from the American Type Culture Collection (ATCC) situated in Manassas, Virginia, United States of America. The HUVECs utilized in this investigation were kindly provided by the Institute for Cardiovascular Science at Soochow University in Suzhou, China. The GES-1 and HGC-27 cell lines were cultivated in Dulbecco’s Modified Eagle Medium (DMEM, EallBio, Beijing, China, #03.1006C), whereas the AGS, MKN-28, and MKN-45 cell lines were cultured in Roswell Park Memorial Institute-1640 Medium (RPMI-1640, EallBio, #03.4007C). EBM-2 media (Lonza, Walkersville, USA, #CC-3162) was used to cultivate HUVECs. The medium employed in this study were supplemented with a 10% concentration of fetal bovine serum (FBS, EallBio, #03.U16001DC) and a 1% concentration of penicillin-streptomycin-amphotericin B (NCM Biotechnology, Suzhou, China, #C100C5). The cells were cultured in a controlled environment using a humidified incubator maintained at a temperature of 37 °C and an atmosphere consisting of 5% CO_2_.

### Cell transfection and lentivirus infection

Two commercially available non-overlapping small interfering RNAs (siRNAs) targeting HOOK3, namely si-HOOK3-1 (5′-AGCGCGAAGTCAACTTGAA-3′) and si-HOOK3-2 (5′-CATCCGTACTTTAGATCCT-3′), along with a control siRNA, were acquired from RiboBio Company (Guangzhou, China). The plasmids for overexpression of HOOK3, VEGFA, and SP1, along with their corresponding control plasmids, were acquired from Wuhan Miaoling Biotechnology Company located in Wuhan, China. The transfection of siRNAs or expression plasmids was performed using Lipo8000™ Transfection Reagent (Beyotime, Shanghai, China) following the guidelines provided by the manufacturer.

The lentiviruses used in this study were purchased from Suzhou GenePharma Co., Ltd. (Suzhou, China). These lentiviruses included a plasmid that overexpressed HOOK3 or contained short hairpin RNA (shRNA) targeting HOOK3 with the si-HOOK-1 sequence. The lentiviruses containing a plasmid that induces the overexpression of VEGFA were acquired from Wuhan Miaoling Biotechnology Company. An empty vector was employed as a negative control. When the cell cultures of AGS, MKN-28, and HGC-27 reached a confluence level of 40%, they were infected with lentiviral particles at a multiplicity of infection (MOI) of 40.

### Total RNA isolation and real-time quantitative polymerase chain reaction (RT-qPCR) assay

The Cell/Tissue Total RNA Kit (Yishan Biotech) was employed in strict adherence to the established methodology for the extraction of total RNA from HGC-27 and MKN-28 cells. The cDNA synthesis procedure involved the utilization of 1.0 μg of total RNA inside a 10 μl reaction volume. Hiscript III qRT SuperMix and dsDNase (Vazyme, Nanjing, China) were employed for this purpose. The heat cycling conditions used during the process were as follows: The samples were subjected to a temperature of 37 °C for a duration of 15 min, followed by a temperature of 85 °C for a duration of 5 s. The RT-qPCR was performed using a CFX96 Real-Time PCR System manufactured by Bio-Rad. The AceQ qPCR SYBR Green Master Mix from Vazyme was utilized for the experiment. The qPCR cycling protocol involved an initial denaturation step at 95 °C for 5 min, followed by 40 amplification cycles consisting of a 10-s denaturation step at 95 °C and a 30-s annealing/extension step at 60 °C. The gene selected for normalization in this study was GAPDH. PCR amplification was conducted using three technical replicates. The primers employed for reverse transcription quantitative RT-qPCR can be located in Supplementary Table S1.

### Protein extraction and Western blot analysis

The cells were collected and subjected to lysis using an SDS lysis buffer (Beyotime, #P1045) containing a protease inhibitor (Beyotime, #P0013G). The concentration of protein was measured using the improved BCA protein assay kit (Beyotime, #P0010S). Following this, a total of 30 μg of protein was separated using a 10% SDS-PAGE gel (NCM Biotech, Suzhou, China, #P2012) and subsequently deposited onto a 0.45 µm PVDF membrane (GE Healthcare Life Science, Germany). In order to reduce nonspecific binding, the membrane was subjected to an incubation process involving 5% bovine serum albumin (BSA) obtained from Beyotime (Shanghai, China, #ST2249-5g) for a duration of 1 h. Subsequently, the membrane was incubated overnight at a temperature of 4 °C with the specified primary antibody. The Western blotting procedure employed a selection of primary antibodies, namely HOOK3 (Proteintech, #15457-1-AP), VEGFA (Proteintech, #19003-1-AP), SP1 (Proteintech, #21962-1-AP), and GAPDH (Proteintech, #60004-1-Ig). The same day, the membrane underwent three washes with TBST (1×TBS, 0.1% Tween 20). Subsequently, it was incubated with the suitable secondary antibody at room temperature for a duration of 1 h, followed by two more washes with TBST. The membrane was seen by employing the ECL reagent (Vazyme, Nanjing, China, #E422-02) and the ChemiDocTM MP imaging equipment (Bio-Rad). The antibodies employed for western blot analysis, together with their corresponding dilution conditions, are outlined in Table S3.

### CCK8 assay

The assessment of cell proliferation was conducted utilizing the Cell Counting Kit-8 (CCK-8, NCM Biotech, Suzhou, China, #C6005). The GC cells were seeded into a 96-well plate at a density of 1000 cells per well. In order to augment the development of color, a volume of 10 μl of CCK8 reagent was administered to each well, followed by an incubation period of 2 h at a temperature of 37 °C. The measurement of absorbance was conducted at a specific wavelength of 450 nm.

### EdU incorporation assay

The cells were subjected to overnight growth on a 48-well plate in preparation for the EdU incorporation experiment. Following that, the cells were grown in a medium containing the EdU working solution (Beyotime, #C0075). Following a period of incubation at a temperature of 37 °C for a duration of 2 h, the cells were subjected to fixation using a 4% solution of paraformaldehyde (Beyotime, Shanghai, #P0099). Subsequently, permeabilization of the cells was achieved by treating them with a 0.3% solution of Triton X-100 in phosphate-buffered saline (PBS). After the application of the Click Additive Solution, the cells were subjected to incubation with Hoechst dye (Beyotime, #C1025). Subsequently, the enumeration of positive cells was conducted utilizing a fluorescent microscope.

### Colony formation assay

The cells of GC were cultivated in a 12-well plate, with each well containing a density of 1000 cells, for a period of 14 days. Subsequently, the culture supernatant was extracted, and the cells were immobilized by employing a 4% paraformaldehyde solution. Following that, the cells that had been immobilized were treated with a crystal violet staining solution (Beyotime, #C0121), and photographs were taken using an inverted microscope.

### Transwell migration and invasion assay

In the migration experiment, a volume of 200 μl of serum-free culture medium containing 5 × 10^4^ GC cells was introduced into the top chamber. The upper chamber was equipped with a membrane that had a hole size of 8 μm (Corning, #353097). A volume of 500 μl of culture media supplemented with 20% serum was introduced into the lower chamber. Regarding the invasion assay, the upper chamber’s membrane was coated with Matrigel (diluted at a ratio of 1:30; Corning, #356234). Following this, the cells were subjected to incubation at a temperature of 37 °C for a duration of 24–48 h. The cells situated beneath the membrane of the top chamber were immobilized using a 4% paraformaldehyde solution and afterwards underwent staining with crystal violet (Beyotime, Shanghai, #C0121). Following this, photographs were acquired with an inverted microscope.

### Apoptosis assay

The apoptosis rate was determined by a PE Annexin V Apoptosis Detection Kit I (BD Biosciences, #559763). Cells were collected and washed with PBS. Then, the cells were resuspended in 1 × Binding Buffer at a concentration of 5 × 10^5^ cells/ml. Then, 2.5 µl of Annexin V-PE and 2.5 µl of 7-AAD were added to the cell suspension. After incubation for 20 min at RT in the dark, apoptosis was analysed by flow cytometry. Annexin-V+/7-AAD- cells and Annexin-V+/7-AAD+ cells were considered apoptotic cells.

### RNA sequencing and bioinformatic analysis

The extraction of total RNA was performed on MKN-28 cells that were overexpressing HOOK3, as well as on control cells. This was achieved using the Total RNA Extraction Reagent (Vazyme, #R401-01). Following this, RNA sequencing (RNA-Seq) was performed by Shanghai Biotechnology Co. Ltd, located in Shanghai, China. Differential gene expression was assessed using two particular criteria: (1) a fold change > 1.5, and (2) *P*-value < 0.05. The generation of volcano plots, GO enrichment analysis, and sumcount was performed using the R software. This analysis involved the utilization of specific packages, namely ‘ggplot2’, ‘enrichGO’, and ‘ggrepel’. The genes that exhibit differential expression, referred to as differentially expressed genes (DEGs), can be found in Supplementary Table S2. The correlation analysis is presented in Supplementary Table S3.

### Endothelial tube formation assay

HUVECs were cultured at a cellular density of 4 × 10^4^ cells per well on 96-well plates that had been pre-coated with 50 μl of Matrigel (Corning). Subsequently, the cells were subjected to incubation in a conditioned culture medium for a duration of 3 h at a temperature of 37 °C in an environment containing 5% carbon dioxide. Following that, the tubules that were generated were observed using a microscope and analyzed utilizing the Image-Pro Plus software.

### Promoter prediction analysis

The GRCh38/hg38 human assembly was accessed using the UCSC Genome Browser at the University of California, Santa Cruz (UCSC), where the promoter sequence of the VEGFA gene was obtained. We utilized the JASPAR, TFDB, and GTRD databases in order to make projections concerning potential transcription factors and the binding sites associated with them. Utilizing a Venn diagram viewer called Jvenn allowed for the display of the intersection between these three datasets. The intersection was represented as a circle. On the official JASPAR website, which may be found at https://jaspar.genereg.net/, one can acquire information on the anticipated binding sites.

### Dual-luciferase reporter assay

The Dual-Luciferase Reporter Assay System (Yeasen, Shanghai, China) was utilized in order to evaluate the luciferase activity of the VEGFA promoter. The evaluation was carried out in accordance with the directions that were published. In this study, MKN-28 and HGC-27 cell lines were subjected to co-transfection with the VEGFA luciferase reporter vector (pGL4-VEGFA; GenePharma), along with plasmids overexpressing HOOK3 and SP1. The transfection process was facilitated using Lipo8000™ Transfection Reagent. Following a 48-hour incubation period, the cellular structures were disrupted through the utilization of a lysis solution known as PLB. The luminometer manufactured by Biotek was utilized to quantify the activity of both firefly and Renilla luciferases. The relative luciferase activity was determined by dividing the firefly luciferase activity by the Renilla luciferase activity, and afterwards normalizing it to OE-NC.

### Chromatin immunoprecipitation (ChIP) assay

The ChIP assay was conducted using the BeyoChIP™ ChIP Assay Kit (Beyotime) in accordance with a previously documented methodology. In summary, the cells OE-NC and OE-HOOK3 MKN-28 were subjected to a 10-minute treatment with 37% formaldehyde in order to induce crosslinking of the chromatin. The chromatin was subjected to sonication using suitable ultrasonic parameters (performed on ice; at 10% power; with a 3-s sonication followed by a 10-s pause; repeated 16–18 times) in order to generate DNA fragments spanning a range of 100–1000 base pairs in length. The immunoprecipitation procedure was conducted utilizing a rabbit anti-SP1 antibody obtained from Proteintech. As a negative control, normal rabbit IgG was employed. Following the de-crosslinking of the complexes, the purified chromatin underwent PCR amplification with specialized primers that were created specifically for the VEGFA promoter region. The DNA fragments obtained were subsequently subjected to analysis using agarose gel electrophoresis. Please see Supplementary Table S1 for the primer sequences.

### Xenograft tumor model

Female mice of the NSG strain, were obtained from the Shanghai Experimental Animal Center for the purpose of the study. The mice had an average age of 5 weeks. A randomization method was used to divide the NSG mice into two groups: the OE-HOOK3 group and the OE-NC group. Both groups were given the same number of animals. There were four mice in each of the groups. To generate a xenograft tumor model, a total of 1 × 10^7^ OE-HOOK3 MKN-28 cells or OE-NC MKN-28 cells were injected subcutaneously into the central flank area of the mice. This was done so that the tumor could grow in the animals. The volume of the tumor was measured using a digital caliper at regular intervals of two days, and then the growth rate was computed thereafter. These measurements were carried out at regular intervals. After the euthanasia of all of the animals, the tumor masses were eventually made available for analysis after a period of 14 days had passed. In addition to that, IHC examination was performed on the tumor tissues.

### Murine lung metastasis model

The NSG mice were randomly allocated into two groups: the OE-HOOK3 group and the OE-NC group. Every group was comprised of three mice. The mice belonging to the OE-HOOK3 group were subjected to intravenous injections of MKN-28 cells that demonstrated an increased expression of HOOK3. On the other hand, the mice belonging to the OE-NC group were administered injections of MKN-28 cells that contained a vector lacking any genetic material. Each mouse was administered a dosage of 3 × 10^6^ cells in a 200 μl solution of phosphate-buffered saline (PBS). After a period of 6 weeks, the mice were euthanized following the delivery of shots. Following that, the lungs were extracted and underwent fixation by immersion in a solution composed of 4% paraformaldehyde. The lung tissues that had been stabilized were treated with hematoxylin and eosin staining. Two proficient pathologists independently performed a quantification of the lung metastatic nodules.

### TUNEL assay

The apoptosis of tumor was detected in vivo using the One Step TUNEL Apoptosis Assay Kit (Beyotime, Shanghai, China, #C1086) strictly in accordance with the kit instructions. The TUNEL-positive tissues emit green fluorescence. DAPI (Beyotime, Shanghai, China, #C1005) was used for nuclear staining. The TUNEL-positive neurons were observed and photographed under the fluorescence microscope.

### Statistics

The data that were gathered were analyzed using GraphPad Prism (version 9.0) for statistical purposes. The application of the Student’s *t*-test was limited to data that exhibited a normal distribution, while the utilization of the Wilcoxon rank-sum test was reserved for data that did not satisfy the assumptions of normality. The study employed the Kaplan-Meier method to do survival analysis. A statistical significance level of less than 0.05 was deemed to be significant.

### Supplementary information

supplementary Fig. 1

supplementary Fig. 2

supplementary Fig. 3

Supplementary Table 1

Supplementary Table 2

Supplementary Table 3

Supplementary figure and table legend

aj-checklist

## Data Availability

The authors declare that all data generated or analyzed during this study are included in this published article. The data presented in this study are available on request from the corresponding authors.
