# Supplementary figures and images for: HOOK3 suppresses proliferation and metastasis in gastric cancer via the SP1/VEGFA axis
Source: Cell Death Discov. 2024 Jan 16;10:33. doi: 10.1038/s41420-024-01808-8 (PMC10791617; doi:10.1038/s41420-024-01808-8)

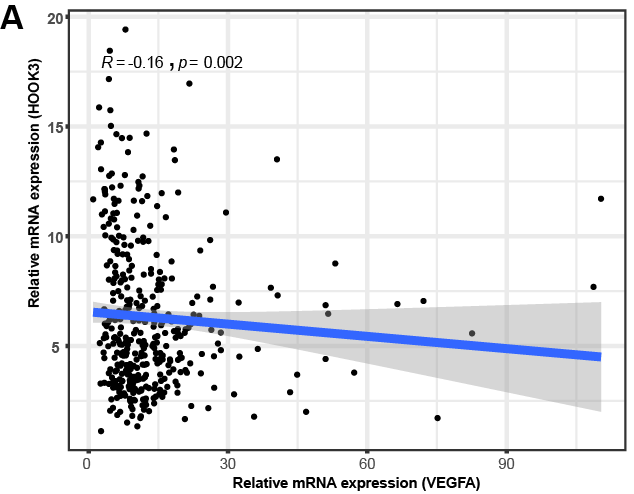

Supplement: Supplementary file 1 — supplementary Fig. 1 [file 41420_2024_1808_MOESM1_ESM.png]

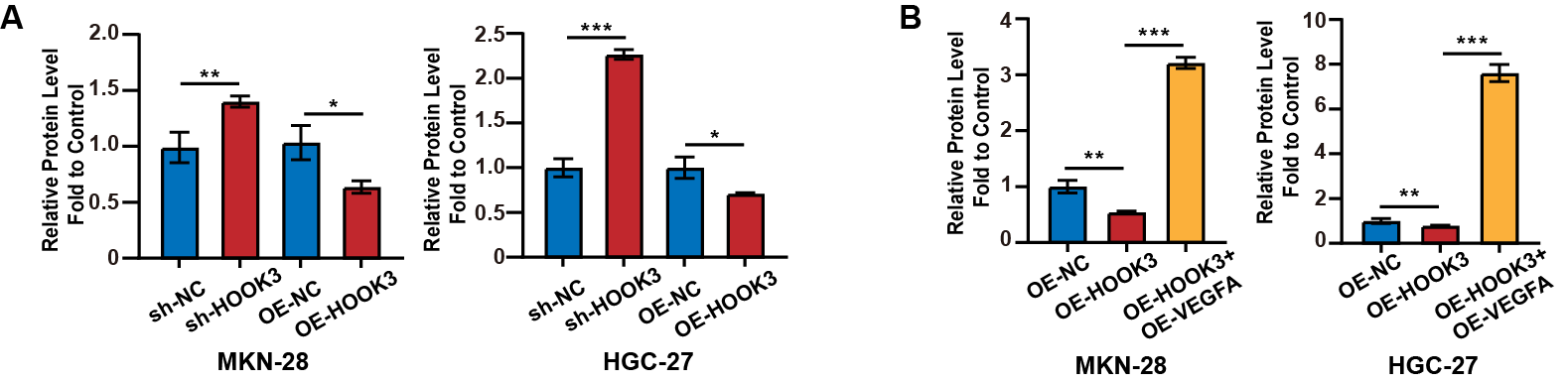

Supplement: Supplementary file 2 — supplementary Fig. 2 [file 41420_2024_1808_MOESM2_ESM.png]

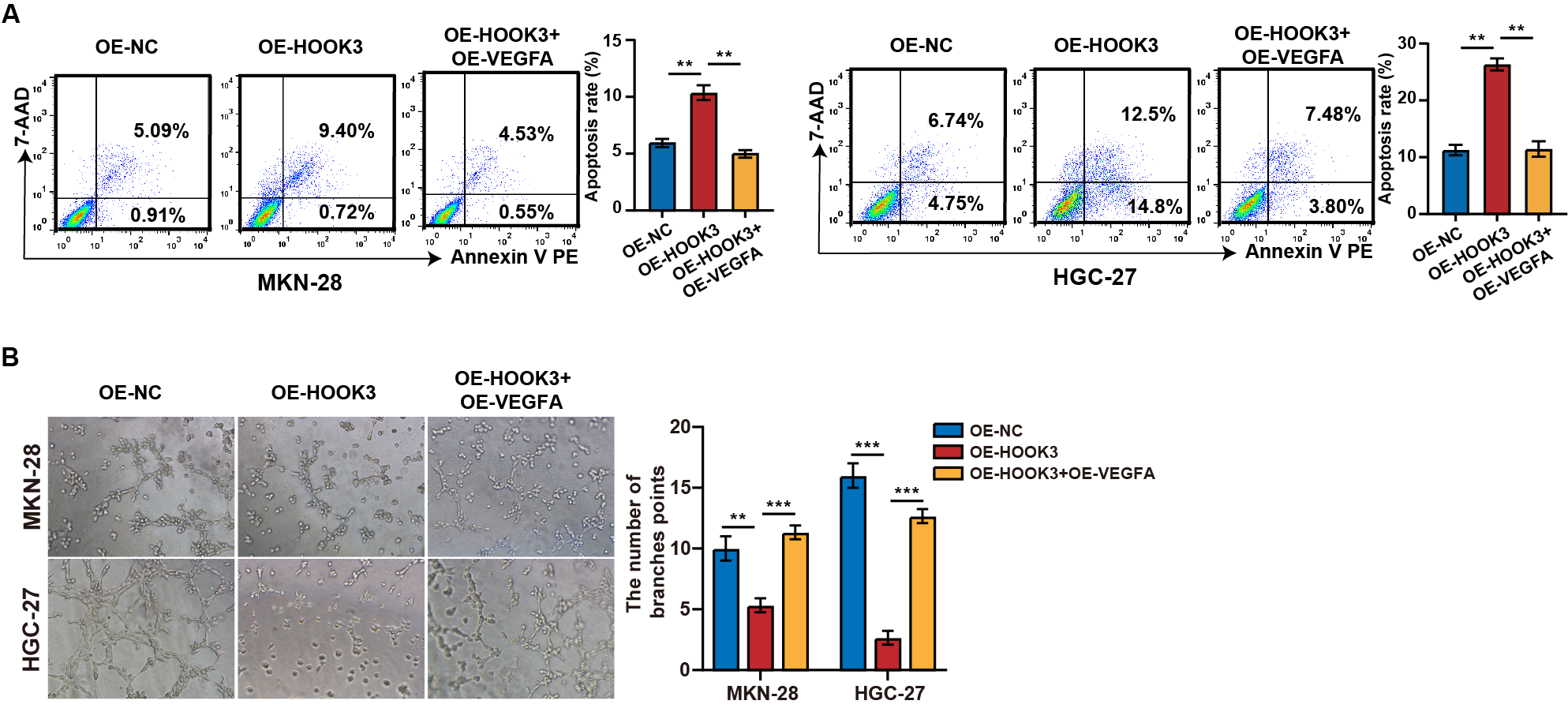

Supplement: Supplementary file 3 — supplementary Fig. 3 [file 41420_2024_1808_MOESM3_ESM.png]
