# Supplementary material for: HOOK3 suppresses proliferation and metastasis in gastric cancer via the SP1/VEGFA axis
Source: Cell Death Discov. 2024 Jan 16;10:33. doi: 10.1038/s41420-024-01808-8 (PMC10791617; doi:10.1038/s41420-024-01808-8)
Supplement: Supplementary file 4 — Supplementary Table 1 [file 41420_2024_1808_MOESM4_ESM.docx]

**Table S1** Primers utilized for RT-qPCR and PCR

| Gene | Forward (5’ to 3’) | Reverse (5’ to 3’) |
| --- | --- | --- |
| GAPDH | GGAGCGAGATCCCTCCAAAAT | GGCTGTTGTCATACTTCTCATGG |
| YY1 | AAGAGCGGCAAGAAGAGTTAC | CAACCACTGTCTCATGGTCAATA |
| SP1 | GTGGAGGCAACATCATTGCTG | GCCACTGGTACATTGGTCACAT |
| ZEB1 | TTACACCTTTGCATACAGAACCC | TTTACGATTACACCCAGACTGC |
| P1 (-367/-358) | AGATCTGTGTGTCCCTCTCCC | GCTAGGAATATTGAAGGGGGCA |
| P2 (-642/-632) | AGGTCAGAAATAGGGGGTCCA | TGGCTAAAGAGGGAATGGGC |
| P3 (-76/-68) | GGCTGAGGCTCGCCTGT | CCGCTACCAGCCGACTTTT |
