# Supplementary material for: HOOK3 suppresses proliferation and metastasis in gastric cancer via the SP1/VEGFA axis
Source: Cell Death Discov. 2024 Jan 16;10:33. doi: 10.1038/s41420-024-01808-8 (PMC10791617; doi:10.1038/s41420-024-01808-8)
Supplement: Supplementary file 7 — Supplementary figure and table legend [file 41420_2024_1808_MOESM7_ESM.docx]

**Supplementary Fig. 1 The relationship between HOOK3 and VEGFA in GC tissues**

**A** Correlation analysis (R=0.16, *P*=0.002) between HOOK3 and VEGFA at the mRNA level in GC tissues in the TCGA database

**Supplementary Fig. 2 HOOK3 modulated VEGFA expression in GC cells**

**A** The Western blot band densities in Figure 4D were quantified using the ImageJ program.

**B** The Western blot band densities in Figure 4E were quantified using the ImageJ program. The experiments were performed in triplicate.

The values were reported as means with standard deviation (SD), and statistical significance was evaluated using Student's t-test.*P < 0.05, **P < 0.01, and ***P < 0.001.

**Supplementary Fig. 3 HOOK3 overexpression inhibited survival of GC cells and tube formation of endothelial cells via VEGFA**

**A** The apoptosis assays were performed on HOOK3-overexpressing GC cells treated with a VEGFA overexpression plasmid.

**B** The tube formation assay was performed on HUVECs treated with conditioned medium obtained from HOOK3-overexpressing MKN-28 and HGC-27 cells transfected with a VEGFA overexpression plasmid.

The experiments were conducted in triplicate. The results were reported as means with standard deviation (SD), and statistical significance was determined using the Student's t-test.**P < 0.01, and ***P < 0.001.

**Supplementary Table 1** Primers utilized for RT-qPCR and PCR

**Supplementary Table 2** Differentially expressed genes in RNA-seq

**Supplementary Table 3** Negative correlation analysis
